# PD-1/PD-L1 inhibitors for early and middle stage microsatellite high-instability and stable colorectal cancer: a review

**DOI:** 10.1007/s00384-024-04654-3

**Published:** 2024-05-29

**Authors:** Huiming Wu, Min Deng, Dingwen Xue, Renkai Guo, Chenyu Zhang, Jiaqi Gao, Huiyu Li

**Affiliations:** Department of General Surgery, Third Hospital of Shanxi Medical University, Shanxi Bethune Hospital, Shanxi Academy of Medical Sciences, Tongji Shanxi Hospital, Taiyuan, China

**Keywords:** Programmed cell death receptor 1, Programmed cell death ligand 1, Microsatellite high-instability, Microsatellite stable, Colorectal cancer

## Abstract

**Background:**

Programmed cell death receptor 1 (PD-1) and programmed cell death ligand 1 (PD-L1) are important immune checkpoint molecules that contribute to tumor immune evasion. However, the main treatment modalities for patients with early and intermediate stage colorectal cancer (CRC) are surgery, and the role of PD-1/PD-L1 inhibitors in these patients is not yet clear. Therefore, this study aims to review the treatment progress of PD-1/PD-L1 inhibitors for early- and intermediate-stage microsatellite high-instability (MSI-H) and stable (MSS) colorectal cancer, in order to provide more options for patients with early- and intermediate-stage colorectal cancer.

**Materials and methods:**

A scoping review of clinical trial registries (Clinicaltrials.gov and EU clinical trial registers) and PubMed/Medline database of trials on PD-1/PD-L1 Inhibitors for early and middle-stage MSI-H and MSS CRC was done up to March 2024.

**Results:**

A total of 19 trials related to early to mid-stage MSH-I or MSS CRC were included. Among them, 6 trials are in recruiting status, 3 trials are in active, not recruiting status, 3 trials are completed, 1 trial is terminated, and 1 trial is unknown. Of these, 9 trials involve MSI-H type CRC, and 10 trials involve MSS type CRC. Preclinical phase I/II trials are predominant, with only 3 clinical phase III trials. In trials related to MSI-H type CRC, 4 studies involve PD-1/PD-L1 inhibitors combined with neoadjuvant therapy, and 5 studies involve combination therapy. In trials related to MSS type CRC, 3 studies involve PD-1/PD-L1 inhibitors combined with targeted therapy, 2 studies involve PD-1/PD-L1 inhibitors combined with chemotherapy, 1 study involves PD-1/PD-L1 inhibitor combined immunotherapy, 1 study involves PD-1/PD-L1 inhibitors combined with bacterial therapy, and 3 studies involve PD-1/PD-L1 inhibitors combined with comprehensive therapy. As for primary outcome measures, 4 trials select pathological complete response rates, 3 trials select progression-free survival rate, 3 trials select objective response rate, 3 trials select overall survival rate, 4 trials select disease-free survival rate, 1 trial selects clinical complete response rate, and 1 trial selects percentage of participants with a dose-limiting toxicity.

**Conclusion:**

For early- and middle-stage MSI-H and MSS CRC, PD-1/PD-L1 inhibitors have shown some therapeutic efficacy, as evidenced by phase I/II studies. However, contemporary trial designs exhibit heterogeneity, with relatively few inclusion criteria, the use of various drug combinations and regimens, and significant variations in reported endpoints. Nevertheless, more double-arm, multicenter, randomized controlled trials are still needed to confirm the efficacy of immunotherapy.

## Introduction

Colorectal cancer (CRC) ranks among the most prevalent and deadly malignancies globally, holding the third and second positions, respectively [1, 2]. In recent years, PD-1/PD-L1 inhibitors have swiftly emerged as a principal therapeutic modality for numerous solid tumors owing to their remarkable efficacy [3]. PD-1 and PD-L1 are pivotal immune checkpoint molecules implicated in tumor immune evasion. By obstructing the PD-1/PD-L1 pathway, the reactivation of cytotoxic T cells against tumor cells is facilitated. Presently, PD-1/PD-L1 inhibitors have garnered approval from the US Food and Drug Administration (FDA) for treating various solid tumors. Studies suggest a close association between the occurrence and progression of CRC and the immune escape mediated by the PD-1/PD-L1 signaling pathway, rendering PD-1 and PD-L1 the most sought-after targets in immunotherapy [4]. Microsatellites are DNA repetitive sequences comprised of 1 to 6 nucleotides. Due to variability in repeat numbers within their core regions, microsatellites are prone to insertions or deletions during replication, resulting in microsatellite instability (MSI) [5]. Mismatch repair (MMR) is the primary DNA repair mechanism targeting such replication errors. MMR deficiency (dMMR) and proficient MMR (pMMR) correspond to MSI-H and MSI-L/MSS, respectively, exhibiting similar biological features. Studies have indicated a higher prevalence of MSI in CRC, with approximately 10 to 15% of CRC patients being MSI-H. MSI-H CRC patients typically exhibit poorer differentiation, mucinous histology, and pronounced lymphocytic infiltration in and around tumors, correlating with elevated PD-L1 expression levels [6–8]. This underscores the heightened potential for MSI-H colorectal cancer patients to benefit from PD-1 inhibitors, yielding superior survival outcomes [9]. However, current standard treatments for early- and intermediate-stage CRC predominantly encompass surgery, radiotherapy, chemotherapy, and anti-angiogenesis therapy [10]. The therapeutic role of immune checkpoint inhibitors in these patients remains uncertain, particularly in cases of MSS colorectal cancer, where studies suggest minimal efficacy of single immune checkpoint inhibitors. Therefore, this review aims to elucidate the therapeutic advancements and strategies involving PD-1/PD-L1 inhibitors in early- and intermediate-stage microsatellite-high and stable colorectal cancer, with the objective of offering broader therapeutic options for patients in these stages.

## Methods

### Search strategy

References for this review were identified through searches of PubMed and clinical trial registries with the medical subject heading (MeSH) search terms: “Colorectal cancer AND Immunotherapy", “Colon cancer AND Immunotherapy", “Rectal cancer AND Immunotherapy", “Colorectal cancer AND Microsatellite Instability", “Colon cancer AND Microsatellite Instability", “Rectal cancer AND Microsatellite Instability", from 2014 (FDA approval of pembrolizumab in September 2014).

We conducted searches on www.ClinicalTrials.gov and the EU Clinical Trials Register using the search phrases “Colorectal cancer AND Immunotherapy", “Colon cancer AND Immunotherapy", “Rectal cancer AND Immunotherapy", “Colorectal cancer AND Microsatellite Instability", “Colon cancer AND Microsatellite Instability", and “Rectal cancer AND Microsatellite Instability", Additionally, we searched the available English literature in PubMed/Medline using the same medical subject heading (MeSH) terms, either individually or in combination, to identify trials, study protocols, and abstracts.

### Eligibility criteria and study selection

Two independent authors (HM W, M D) meticulously sifted through articles retrieved from the initial literature search, meticulously removing duplicate studies and excluding those not directly pertinent to the research. The two authors then independently conducted a detailed review of studies that met the predetermined eligibility criteria, whether in abstract or full-text form, carefully evaluating their alignment with the specified standards. Any disparities in study selection were methodically addressed through thorough discussions, consensus-building, or seeking input from a third independent author (HY L). The inclusion criteria were thoughtfully outlined as follows: (1) Male or female subjects aged ≥ 18 years, (2) locally confirmed dMMR/MSI-H or pMMR/MSS colorectal carcinoma; (3) all subjects were clinically and pathologically confirmed or diagnosed through laboratory examinations as early and middle stage colorectal cancer; (4) all patients received PD-1/PD-L1 therapy; (5) the research methodology is adequately described and scientifically reliable; (6) articles identified as editorials, letters, case reports, or case series were excluded.

## Result

### Literature searching

**Fig. 1:**
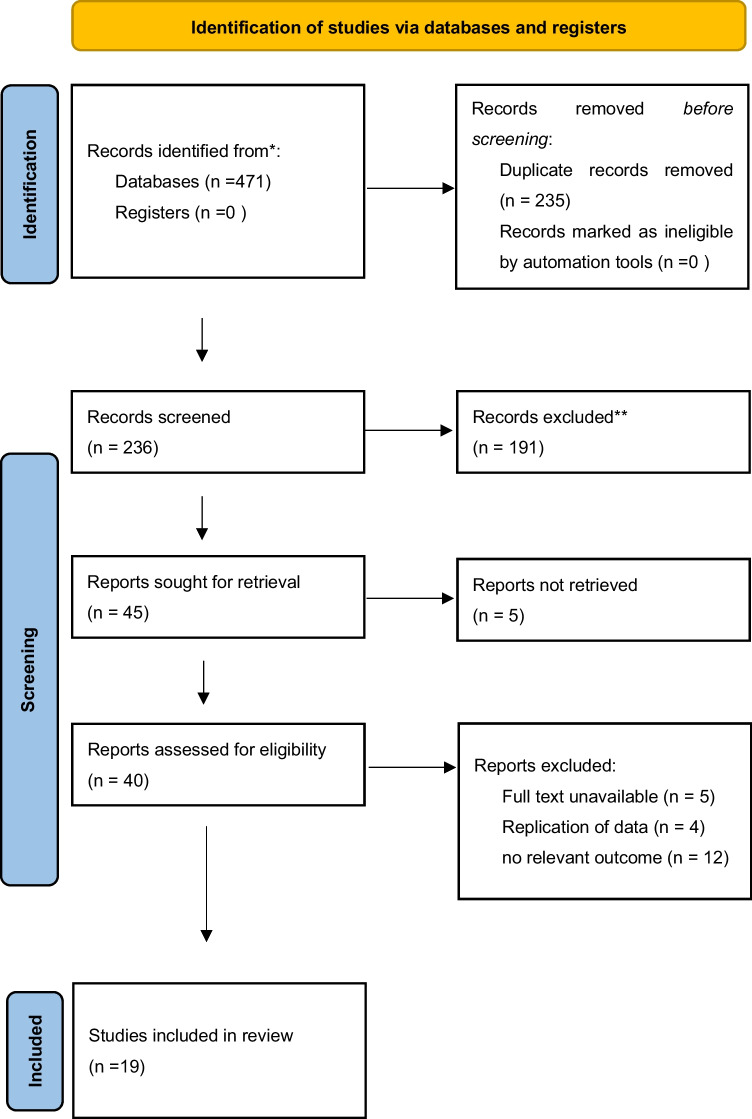
PRISMA flow chart of study selection. *Consider, if feasible to do so, reporting the number of records identified from each database or register searched (rather than the total number across all databases/registers). **If automation tools were used, indicate how many records were excluded by a human and how many were excluded by automation tools. From: Page MJ, McKenzie JE, Bossuyt PM, Boutron I, Hoffmann TC, Mulrow CD, et al. The PRISMA 2020 statement: an updated guideline for reporting systematic reviews. BMJ 2021;372:n71. https://doi.org/10.1136/bmj.n71

During the initial screening, a total of 471 trials were identified. After removing duplicates, we screened 236 trials and identified 45 eligible trials by reviewing titles and abstracts. Among these 45 trials, 19 trials were determined to meet the inclusion criteria for the final analysis after a full-text evaluation. The study selection progress is presented in the PRISMA flow diagram (Fig. 1).

### Characteristics of the included trials

A total of 19 trials related to early to mid-stage MSH-I or MSS CRC were included. Among them, 6 trials are in recruiting status, 3 trials are in active, not recruiting status, 3 trials are completed, 1 trial is terminated, and 1 trial is unknown. Of these, 9 trials involve MSI-H type CRC, and 10 trials involve MSS type CRC. Preclinical phase I/II trials are predominant, with only 3 clinical phase III trials. In trials related to MSI-H type CRC, 4 studies involve PD-1/PD-L1 inhibitors combined with neoadjuvant therapy, and 5 studies involve combination therapy. In trials related to MSS type CRC, 3 studies involve PD-1/PD-L1 inhibitors combined with targeted therapy, 2 studies involve PD-1/PD-L1 inhibitors combined with chemotherapy, 1 study involves PD-1/PD-L1 inhibitor combined immunotherapy, 1 study involves PD-1/PD-L1 inhibitors combined with bacterial therapy, and 3 studies involve PD-1/PD-L1 inhibitors combined with comprehensive therapy. As for primary outcome measures, 4 trials select pathological complete response rates, 3 trials select progression-free survival rate, 3 trials select objective response rate, 3 trials select overall survival rate, 4 trials select disease-free survival rate, 1 trial selects clinical complete response rate, and 1 trial selects a percentage of participants with a dose-limiting toxicity. The characteristics of the included trials are summarized in Table 1.

**Table 1:** Characteristics of the included studies

Study	Country	Phase	Enrollment	Patient condition	Intervention/treatment	Primary outcome measures	Research status
NCT02870920	Canada	II	180	pMMR/MSS CRC	Tremelimumab + durvalumab	Overall survival rate	Completed
NCT02912559	USA	III	700	Stage III dMMR / MSI-H CRC	Chemotherapy alone or combined with atezolizumab as adjuvant therapy	Disease-free survival rate	Active, not recruiting
NCT02948348	Japan	II	90	pMMR/MSS CRC	Nivolumab + ipilimumab	Overall survival rate	Unknown
NCT03374254	USA	I	114	pMMR/MSS CRC	Pembrolizumab + mFOLFOX or FOLFIRI	Percentage of participants with a dose limiting toxicity	Active, not recruiting
NCT03388190	Norway	II	80	Locally-advanced or Metastatic pMMR/MSS CRC	Nivolumab + FLOX	Progression-free survival rate	Recruiting
NCT03721653	Italy	II	218	Locally-advanced or Metastatic pMMR/MSS CRC	FOLFOXIRI plus bevacizumab with or without atezolizumab	Progression-free survival rate	Completed
NCT03827044	UK	III	30	Stage III dMMR / MSI-H CRC	Avelumab plus 5-FU-based chemotherapy	Disease-free survival rate	Terminated
NCT03854799	Italy	II	101	Locally-advanced pMMR/MSS CRC	Avelumab + capecitabine + radiation	Pathological complete response rates	Completed
NCT03926338	China	II	34	Surgically resectable non-metastatic dMMR/MSI-H CRC	Tremelimumab + ipilimumab VS tremelimumab	Pathological complete response rates	Completed
NCT04258111	China	II	4	Locally-advanced or Metastatic dMMR / MSI-H CRC	IBI310 + sintilimab	Objective response rate	Active, not recruiting
NCT04561336	Italy	II	77	pMMR/MSS CRC	Avelumab + cetuximab	Overall survival rate	Completed
NCT04715633	China	II	53	Locally Advanced dMMR/MSI-H CRC	Camrelizumab + apatinib	Clinical complete response rate	Recruiting
NCT05116085	China	II	33	early-stage (Stage II-III) MSI-H/dMMR CRC	Tislelizumab	Major pathological response rate	Recruiting
NCT05118724	Germany	II	80	Stage III dMMR / MSI-H CRC	Atezolizumab	Disease-free survival rate	Recruiting
NCT05236972	China	III	323	Stage III dMMR / MSI-H CRC	Sintilimab vs standard therapy	Disease-free survival rate	Recruiting
NCT05371197	China	II	26	Surgically resectable non-metastatic dMMR/MSI-H CRC	Neoadjuvant therapy with PD-L1 inhibitor	Pathological complete response rates	Recruiting
REGNIVO37	USA	II	70	Surgically resectable MSS CRC	Regorafenib + nivolumab	Objective response rate	Completed
REGONIVO36	Japan	I	25	Surgically resectable MSS CRC	Nivolumab + regorafenib	Objective response rate	Completed
RENMIN-25	China	II	20	pMMR/MSS CRC	Fecal microbiota transplantation + tislelizumab + fruquintinib	Progression-free survival/overall survival rate	Completed

### Treatment strategy for early to mid-stage MSI-H CRC with PD-1/PD-L1 inhibitorss

#### Combined with neoadjuvant therapy

Neoadjuvant therapy involves administering chemotherapy and combination therapy before surgical treatment to reduce tumor staging, decrease disease recurrence, and achieve a better prognosis. The standard treatment regimen for early- to intermediate-stage CRC patients typically involves postoperative chemotherapy or observation without chemotherapy. However, the risk of postoperative recurrence remains significant. In recent years, neoadjuvant therapy not only reduces the risk of tumor recurrence but also allows some patients with previously inoperable tumors to have the opportunity to have their tumors resected after treatment [11, 12]. The advent of the immunotherapy era has opened a new chapter in neoadjuvant immunotherapy.

In 2015, American researchers, led by Le and colleagues [13], conducted independent studies on three patient cohorts and found that MSI-H-type patients responded well to immune checkpoint inhibitors. This suggests that MSI-H patients may benefit from immunotherapy, marking the beginning of a new era in immunotherapy for CRC patients. However, neoadjuvant immunotherapy for early- to intermediate-stage CRC patients is still under exploration. In 2022, a single-center, randomized, phase II trial (NCT03926338) published in The Lancet allocated 34 resectable, locally advanced CRC patients to receive either toripalimab combined with camrelizumab or toripalimab alone before undergoing surgical resection. By August 10, 2021, the pathological complete response (pCR) rate in the experimental group reached 88%, compared to only 65% in the control group. All patients remained alive without recurrence. Regarding the occurrence of treatment-related adverse events, 10 patients in each group experienced grades 1 to 2 treatment-related adverse events, indicating good overall tolerability. This suggests that neoadjuvant toripalimab combined with camrelizumab may become a treatment option for MSI-H locally advanced CRC patients. Although these studies had small sample sizes, they demonstrated remarkable outcomes of neoadjuvant immunotherapy in MSI-H patients [14].

In addition to neoadjuvant immunotherapy combined with chemotherapy, there are other neoadjuvant immunotherapy modalities under investigation, such as neoadjuvant immunotherapy monotherapy and immunotherapy combined with VEGF inhibitors. An open-label, multicenter, phase II clinical trial (NCT04715633) evaluating the PD-1 inhibitor camrelizumab combined with the VEGF inhibitor apatinib for the treatment of locally advanced CRC with dMMR/MSI-H is ongoing. This trial plans to enroll 52 patients, with the primary endpoint being a clinical complete response (cCR) or pathological complete response (pCR), and the secondary endpoint being the objective response rate (ORR). The final results have not yet been disclosed. Two other phase II trials for resectable, locally advanced CRC with dMMR/MSI-H are investigating neoadjuvant immunotherapy with envafolimab monotherapy (NCT05371197) and tislelizumab monotherapy (NCT05116085). The NCT05371197 study aims to recruit 26 patients, with the primary endpoint being pCR, and secondary endpoints including major pathological response rate (MPR), disease-free survival (DFS), and overall survival (OS). The trial commenced in May 2022. NCT05116085 evaluates the safety and efficacy of tislelizumab neoadjuvant therapy in patients with MSI-H/dMMR stage II-III CRC, with the primary endpoint being MPR and the secondary endpoint being pCR.

#### Combination therapy

In early- and mid-stage CRC patients, surgical resection is the primary treatment modality. Adjuvant therapies such as chemotherapy, radiotherapy, and anti-angiogenic therapy following surgery often lead to greater survival benefits and reduce the recurrence rate. For patients who are not eligible for surgical resection, systemic therapies such as adjuvant treatments become particularly important. However, the efficacy of immunotherapy as an adjunctive treatment in combination therapy is yet to be determined, and related studies are still ongoing, with results pending. Studies such as POLEM, ATOMIC, NCT04258111, NCT05236972, and NCT05118724 are currently underway. The POLEM study includes stage III dMMR/MSI-H CRC patients, comparing the efficacy of adjuvant standard chemotherapy combined with PD-1 monoclonal antibodies versus chemotherapy alone [15]. The ATOMIC study compares the efficacy of FOLFOX with or without atezolizumab in stage III dMMR/MSI-H CRC patients. Both studies are ongoing. NCT04258111 is a multicenter, open-label, phase II trial investigating the efficacy of sintilimab combined with IBI310 in locally advanced dMMR/MSI-H CRC patients, with the primary endpoint being ORR and secondary endpoints including PFS, DCR, and DOR. A randomized phase III trial (NCT05236972) explores the effectiveness of sintilimab versus standard therapy in locally advanced dMMR/MSI-H CRC patients, with DFS as the primary endpoint and OS as the secondary endpoint, planning to enroll 323 patients. NCT05118724 analyzes the efficacy of atezolizumab versus atezolizumab combined with IMM-101 (a suspension of heat-killed *Mycobacterium obuense*, which induces CD8+ T cell responses) in dMMR/MSI-H stage III CRC patients who do not qualify for or refuse oxaliplatin-based adjuvant chemotherapy, with plans to enroll 120 patients, with DFS as the primary endpoint and OS as the secondary endpoint. These immunotherapy combination therapies’ clinical studies are currently in the trial phase, and results are awaited. Although the results of these studies have not yet been published, they may open up a new avenue of adjuvant treatment for early and mid-stage CRC patients undergoing surgery or ineligible for surgery, leading to greater survival benefits.

### Treatment strategy for early to mid-stage MSS CRC with PD-1/PD-L1 inhibitors

#### Combination therapy of PD-1/PD-L1 inhibitors with targeted therapy

In CRC treatment, VEGF and epidermal growth factor receptor (EGFR) are common therapeutic targets [16]. Studies have shown that combining immunotherapy with VEGF antagonists and EGFR antagonists may offer new treatment options and hope for pMMR/MSS CRC patients.

VEGF inhibitors reduce the number of tumor neo angiogenesis, promote vascular normalization, and increase oxygen supply, thereby facilitating effective activation and initiation of T cells, synergizing with tumor immunotherapy. The Japanese REGONIVO study first reported an exploratory phase Ib study of nivolumab in combination with regorafenib for refractory MSS CRC, demonstrating an objective response rate (ORR) of 33.3%, with a median progression-free survival (PFS) of 7.9 months, and 1-year PFS and overall survival (OS) rates of 41.8% and 68.0%, respectively, highlighting the benefits of immunotherapy for “cold” CRC patients [17]. However, subsequent studies using different VEGF receptor inhibitors, tyrosine kinase inhibitors (TKIs), in combination with immune checkpoint inhibitors as salvage therapy for CRC patients, such as the North American REGNIVO study [18], have shown some degree of benefit, but none have achieved the high ORR level of the Japanese REOGNIVO study.

Anti-EGFR monoclonal antibodies not only have the ability to directly kill tumor cells but also possess immune-modulatory properties. They can enhance the antibody-dependent cell-mediated cytotoxicity (ADCC) induced by natural killer cells, recruiting CD3+, CD8+, and CD56+ cells to the tumor core, enhancing the immune response within the tumor [19]. Additionally, EGFR monoclonal antibodies can induce PD-L1 expression, enhancing the effectiveness of immunotherapy [20]. Therefore, anti-EGFR monoclonal antibodies have potential synergistic effects with immune checkpoint inhibitors, and their combined use can stimulate both innate and adaptive immune systems to kill tumor cells. The phase II single-arm CAVE trial (NCT04561336) investigated the efficacy and safety of cetuximab rechallenge combined with avelumab in RAS wild-type CRC patients, showing a median OS of 11.6 months and a median PFS of 3.6 months, suggesting that this combination therapy is effective and well-tolerated, and plasma ctDNA analysis before treatment can identify potentially beneficial patients [21]. However, current small-scale study results are insufficient to draw definitive conclusions about the complete effectiveness and widespread applicability of combined targeted therapy strategies in mCRC treatment, requiring further large-sample randomized phase III trials to validate the effectiveness of combination therapy.

#### PD-1/PD-L1 inhibitors combined with chemotherapy

Chemotherapy drugs can enhance the recognition and presentation of dendritic cells (DCs), activate cytotoxic T lymphocytes to attack tumors, stimulate the release of interleukin-2 (IL-2), IL-4, and interferon-gamma (IFN-γ), and induce anti-tumor immune responses [22]. The combination of chemotherapy and immunotherapy can enhance immunogenicity, improve the efficacy of immunotherapy drugs, and suppress chemotherapy resistance. In a phase Ib clinical study, Keynote-651 (NCT03374254), combining pembrolizumab with mFOLFOX or FOLFIRI for first- or second-line treatment of pMMR/MSS CRC patients, demonstrated promising results. The objective response rate (ORR) was 58% for Cohort B (pembrolizumab + mFOLFOX) and 15.6% for Cohort D (pembrolizumab + FOLFIRI), with disease control rates (DCR) of 94% and 63%, respectively, suggesting the safety and efficacy of the combination therapy [23]. Another phase 2 study, METIMMOX (NCT03388190), evaluated the efficacy of oxaliplatin (FLOX) in combination with nivolumab compared to FLOX alone in first-line treatment of MSS CRC patients. The results showed that the combination group had a median progression-free survival (mPFS) of 6.6 months, with an ORR of 46.3% at 8 months, indicating that FLOX could convert MSS to an immunogenic state and increase the likelihood of a response to immune checkpoint inhibitors in this patient population [24]. Chemotherapy combined with immunotherapy has been a common clinical approach, but further research is needed to achieve optimal combination therapy outcomes.

#### Combination of PD-1/PD-L1 inhibitors with radiotherapy

Radiotherapy can synergize with immunotherapy by stimulating the release of pro-inflammatory factors and infiltration of immune cells. Radiotherapy has been shown to reshape the TME through three different mechanisms: inducing immunogenic cell death (ICD) of tumor cells, upregulating the antigen presentation capacity of major histocompatibility complex I (MHC-I), and directly altering the TME at the radiation site [25]. Radiotherapy induces tumor cell ICD by upregulating the release of damage-associated molecular patterns (DAMPs) [26], relieving hypoxia, and T cell immune suppression, thereby eliciting new anti-tumor responses. Additionally, radiotherapy stimulates the recruitment of dendritic cells (DCs), enhances the expression of MHC-I molecules, and improves their antigen presentation capacity. Radiotherapy also induces the expression and release of chemokines such as CXCL9, CXCL10, and CXCL16 in tumor cells, promoting the migration of effector T lymphocytes to tumor sites, modulating the immune status of the TME, and activating the body’s anti-tumor immune response. Furthermore, radiotherapy can induce systemic immunity through the “abscopal effect” [27]. Therefore, radiotherapy plays a role in enhancing the efficacy of immunotherapy by altering the TME.

#### PD-1/PD-L1 inhibitor combined immunotherapy

Research has shown that dual immunotherapy achieves significant clinical efficacy, primarily because the combination of anti-PD-1 and anti-CTLA-4 increases the infiltration of effector T cells, overcoming the innate tolerance of cold CRC patients to monotherapy immunotherapy [28]. CTLA-4 and PD-1 are both immune checkpoint proteins on T cells. Anti-CTLA-4 can expand the number of T cells in lymphoid organs and tumor tissues, while anti-PD-1 can overcome the inhibition of effector T cells. The phase II CCTGCO.26 study (NCT02870920) used PD-L1 monoclonal antibodies in combination with CTLA-4 monoclonal antibodies to treat patients with advanced refractory CRC (98% of whom were pMMR/MSS) [29]. The study results showed that the median OS of the dual immunotherapy group reached 6.6 months, and the DCR was 22.6%. Further analysis indicated that patients with TMB>28MTs/MB could benefit more from dual immunotherapy. Other studies have shown that compared to monotherapy immunotherapy, dual immunotherapy does not significantly increase toxic adverse reactions. LAG-3, as an important new immune checkpoint, can induce apoptosis of immune cells and reduce cytokine secretion. However, although the efficacy of dual immunotherapy has been confirmed, further exploration is still needed in future clinical practice.

#### PD-1/PD-L1 inhibitors combined with oncolytic virus (OVs) therapy

OVs selectively replicate and amplify within tumor cells, leading to their destruction and the release of tumor-associated antigens (TAAs) and neutrophils, inducing immunogenic cell death (ICD) in tumor cells. Additionally, OVs activate and promote T cell infiltration into the tumor site, thereby improving the tumor microenvironment [30]. OVs can induce tumor cell apoptosis through various pathways. Importantly, the subvirions released upon tumor cell lysis can infect surrounding cells and trigger a cascade reaction to amplify the lysis, increasing the cytotoxic effect on tumor cells over time [31]. During ICD, the release of damage-associated molecular patterns (DAMPs) promotes dendritic cell (DC) maturation and T cell activation. OVs also stimulate the production of CXCL9 and CXCL10, providing critical signals for T cell trafficking. Furthermore, OVs can induce degradation of the tumor stroma, breaking down physical barriers to T cell infiltration [32]. However, using OVs alone may not sustain long-term immune responses and may lead to immune resistance. Combining OVs with immune checkpoint inhibitors not only reduces the immune tolerance of tumor cells but also significantly enhances antitumor immune efficacy [33]. Therefore, research results suggest that the combination of OVs and immune checkpoint inhibitors can increase the infiltration of effector T cells into tumor tissues, partially reversing the non-immunogenic inflammatory microenvironment in “cold tumors” [34].

#### PD-1/PD-L1 inhibitors combined with bacterial therapy

The gut microbiota not only participates in the metabolism of host nutrients and the maintenance of intestinal mucosal integrity but also exhibits characteristics of promoting the maturation of immune cells. Therefore, the gut microbiota plays a crucial role in the development and maintenance of the host immune system [35]. Pathogen-associated molecular patterns of gut microbiota are recognized by Toll-like receptors, activating dendritic cells (DCs) to initiate adaptive immune responses, exerting antitumor effects [36]. Moreover, T cell cross-reactivity induced by microbial antigens may interact with tumor-associated antigens (TAAs), thereby inducing specific antitumor immune responses. For example, the antigen SVY on *Bacteroides fragilis* is homologous to the novel antigen SIY in tumor cells, stimulating cross-reactive T cell responses against tumor cells, indicating that microbial “mimic antigens” can modulate T cells and enhance antitumor immunity [37]. Microbial metabolites also indirectly regulate immunity. For instance, *Akkermansia muciniphila* can increase the levels of CD8+ T cells in tumors by secreting adenosine, subsequently inducing the differentiation and activation of CD4+ T cells and promoting the production of IFN-γ, thus participating in immune regulation and promoting antitumor immune responses [38]. A phase 2 trial (RENMIN-25) in China investigated the efficacy of fecal microbiota transplantation combined with toripalimab and fruquintinib in patients with CRC. The results showed a median progression-free survival (PFS) of 9.6 months, a median overall survival (OS) of 13.7 months, and a disease control rate (DCR) of 95%, highlighting the benefits of immune combined bacterial therapy for cold CRC patients [39]. As a relatively new antitumor drug, the gut microbiota plays a significant role in cancer immunotherapy. However, its immunomodulatory mechanisms are complex and influenced by factors such as gender, age, weight, diet, medication use, genetics, and geographical environment [40]. Therefore, despite the enormous potential of gut microbiota intervention in cancer immunotherapy, further preclinical research is needed to determine the optimal strategy for microbial intervention.

#### PD-1/PD-L1 inhibitors combined with comprehensive therapy

Studies have shown that chemotherapy and targeted therapy combined with immunotherapy can improve the anti-tumor immune response in cold CRC patients. A phase 2 clinical trial (NCT03721653), AtezoTRIBE, used FOLFOXIRI plus bevacizumab with or without atezolizumab as the first-line treatment for MSS CRC. The results indicated that adding atezolizumab prolonged PFS in CRC patients without increasing the incidence of adverse reactions. Another phase 2 clinical trial, BACCI, compared the efficacy of capecitabine plus bevacizumab with or without atezolizumab. The results showed no significant improvement in PFS or OS in the combination with atezolizumab group, but the ORR increased from 4.35 to 8.54% [41].

Neoadjuvant chemoradiotherapy (NCRT) is the standard treatment for locally advanced rectal cancer, and many studies have shown that adding immunotherapy based on NCRT can promote treatment response. For example, a clinical study in Japan (NCT02948348) on immunotherapy consolidation after neoadjuvant radiotherapy achieved a pathological complete response (pCR) rate of 29.7% in MSS CRC patients. An Italian phase 2 clinical trial (NCT03854799) observed a pCR rate of 21.8% in MSS CRC patients with sequential immunotherapy after NCRT. However, there is still controversy over the balance between the immunological benefits and adverse reactions brought by immunotherapy combined with multiple therapies, requiring further research and exploration.

## Discussion

With the continuous deepening of understanding in tumor biology and the mechanisms of tumor immune tolerance, the emergence of immunotherapy has broken the treatment bottleneck for CRC patients. As an emerging therapy, it has been widely used in the clinical treatment of various solid tumors, altering the traditional treatment paradigm. However, the actual benefit for CRC patients from immunotherapy is very limited. Current small-scale research results show that while the application of PD-1/PD-L1 inhibitors alone is essentially ineffective in treating MSS-type CRC patients, immune combination therapy strategies can benefit certain MSS-type CRC patients. This article introduces the research progress of immunotherapy combined with targeted therapy, chemotherapy, radiotherapy, oncolytic viruses, bacterial therapy, and various other therapies. The results all demonstrate a certain degree of benefit. However, further large-sample randomized controlled studies are still needed to verify the effectiveness of combination therapy and explore the optimal combination strategies of immunotherapy to maintain long-term anti-tumor immune responses. While continuing to research and develop immunotherapeutic drugs, there is also an active search for predictive biomarkers to accurately select the beneficiary population of immune combination therapy. Currently, MSI is the only approved biomarker for screening colorectal cancer immunotherapy, while other immunotherapeutic biomarkers have their limitations, requiring substantial clinical research to demonstrate accuracy. Additionally, there is a need to optimize the detection methods of biomarkers, establish uniform judgment criteria, facilitate the stratification of CRC patients, improve patient prognosis and overall survival rates, and promote the development of precise biomarkers and precision therapies in the future.

## Data Availability

No datasets were generated or analysed during the current study.
